# Glycoprotein YKL-40: A potential biomarker of disease activity in rheumatoid arthritis during intensive treatment with csDMARDs and infliximab. Evidence from the randomised controlled NEO-RACo trial

**DOI:** 10.1371/journal.pone.0183294

**Published:** 2017-08-25

**Authors:** Tuija Väänänen, Katriina Vuolteenaho, Hannu Kautiainen, Riina Nieminen, Timo Möttönen, Pekka Hannonen, Markku Korpela, Markku J. Kauppi, Kari Laiho, Oili Kaipiainen-Seppänen, Riitta Luosujärvi, Tea Uusitalo, Toini Uutela, Marjatta Leirisalo-Repo, Eeva Moilanen, on behalf of the NEO-RACo Study Group

**Affiliations:** 1 The Immunopharmacology Research Group, Faculty of Medicine and Life Sciences, University of Tampere and Tampere University Hospital, Tampere, Finland; 2 Department of General Practice, University of Helsinki, Helsinki, Finland; 3 Unit of Primary Health Care, Helsinki University Hospital, Helsinki, Finland; 4 Unit of Primary Health Care, Kuopio University Hospital, Kuopio, Finland; 5 MedCare Ltd, Äänekoski, Finland; 6 Department of Rheumatology, University of Turku and Turku University Hospital, Turku, Finland; 7 Department of Medicine, Jyväskylä Central Hospital, Jyväskylä, Finland; 8 Department of Internal Medicine, Centre for Rheumatic Diseases, Tampere University Hospital, Tampere, Finland; 9 Department of Medicine, Päijät-Häme Central Hospital, Lahti, Finland; 10 Faculty of Medicine and Life Sciences, University of Tampere, Tampere, Finland; 11 Department of Medicine, Kuopio University Hospital, Kuopio, Finland; 12 Rheumatology, University of Helsinki and Helsinki University Hospital, Helsinki, Finland; 13 Department of Medicine, Hämeenlinna Central Hospital, Hämeenlinna, Finland; 14 Department of Medicine, Lapland Central Hospital, Rovaniemi, Finland; VU University Medical Center, NETHERLANDS

## Abstract

**Objective:**

YKL-40, a chitinase-like glycoprotein associated with inflammation and tissue remodeling, is produced by joint tissues and recognized as a candidate auto-antigen in rheumatoid arthritis (RA). In the present study, we investigated YKL-40 as a potential biomarker of disease activity in patients with early RA at baseline and during intensive treatment aiming for early remission.

**Methods:**

Ninety-nine patients with early DMARD-naïve RA participated in the NEO-RACo study. For the first four weeks, the patients were treated with the combination of sulphasalazine, methotrexate, hydroxychloroquine and low dose prednisolone (FIN-RACo DMARD combination), and subsequently randomized to receive placebo or infliximab added on the treatment for further 22 weeks. Disease activity was evaluated using the 28-joint disease activity score and plasma YKL-40 concentrations were measured by immunoassay.

**Results:**

At the baseline, plasma YKL-40 concentration was 57 ± 37 (mean ± SD) ng/ml. YKL-40 was significantly associated with the disease activity score, interleukin-6 and erythrocyte sedimentation rate both at the baseline and during the 26 weeks’ treatment. The csDMARD combination decreased YKL-40 levels already during the first four weeks of treatment, and there was no further reduction when the tumour necrosis factor-α antagonist infliximab was added on the combination treatment.

**Conclusions:**

High YKL-40 levels were found to be associated with disease activity in early DMARD-naïve RA and during intensive treat-to-target therapy. The present results suggest YKL-40 as a useful biomarker of disease activity in RA to be used to steer treatment towards remission.

## Introduction

YKL-40 is an inflammation-associated glycoprotein with a molecular weight of 40 kDa. It belongs to the family of chitinase like proteins but lacks the enzymatic activity of true chitinases. YKL-40 is known also by names chitinase-3-like protein 1 (Chi3-l1), breast regression protein 39 (BRP-39), human cartilage glycoprotein 39, and chondrex. It is expressed in various cell types and increased levels of YKL-40 have been linked to inflammation, tissue remodeling and cancer, but the exact biological activities are yet to be identified [1].

Rheumatoid arthritis (RA) is a chronic autoimmune disease affecting principally the joints, but the mechanisms that trigger the autoimmune responses leading eventually to joint destruction are not fully known. At early phases of the process, exogenous and autologous antigens are presented to T-cells by antigen presenting cells and interestingly, YKL-40 is recognized as a candidate autoantigen [2–6]. Circulating YKL-40 levels have been shown to be higher in RA patients as compared to healthy controls [7–13]. Also, the YKL-40 concentrations in synovial fluid (SF) are higher than those measured in plasma indicating significant intra-articular production [7,11]. Within RA joints, YKL-40 has been recognized as a major secretory protein of articular chondrocytes [14]. Synovial cells, macrophages and neutrophils infiltrating into the RA synovium also produce YKL-40 [1,14–16], and just recently, splenic T-cells have been added to the list of YKL-40 producing cells in RA [17].

In the treatment of RA, the current treat-to-target approach aims for early remission or maximally low disease activity. Biological disease modifying anti-rheumatic drugs (bDMARDs), including tumour necrosis factor-α (TNF-α) inhibitors, are recommended to be commenced with conventional systemic disease-modifying antirheumatic drugs (csDMARDs) if the treatment target is not reached with csDMARDs alone [18]. In RA, assessment of disease activity is based on composite indices, such as the 28-joint disease activity score (DAS28) [19] evaluating the count of tender and swollen joints, inflammation and patient´s assessment of the disease activity. We have reported previously based on the current NEO-RACo trial [20–23] excellent sustained clinical results with treat-to-target approach in patients with early, DMARD-naïve RA by using the intensified Finnish Rheumatoid Arthritis combination (FIN-RACo) treatment. This consists of a combination of three csDMARDs (i.e. sulphasalazine, methotrexate and hydroxychloroquine) and low dose glucocorticoid (GC) following a predefined protocol supplemented with active treatment of inflamed joints with intra-articular GC injections [24]. At 2 years, DAS28 remission was achieved in 82% of the patients [20]. Adding infliximab to the FIN-RACo combination treatment for the first 6 months in a randomized, double-blind and parallel-group manner, induced remission more rapidly, but differences between the treatment groups were not statistical significant at 2 or 5 years follow-up [20,21].

In the search of novel biomarkers, we hypothesized that YKL-40, an inflammatory factor produced mainly by intra-articular tissues, could reflect disease activity and inflammation in RA patients. In the present study, we tested this hypothesis by measuring the YKL-40 plasma levels in DMARD-naïve patients at the baseline and during intensive anti-rheumatic treatment in the NEO-RACo study.

## Methods

### Study design, patients, outcomes and follow-up

Ninety-nine (99) patients with early active RA fulfilling the classification criteria positioned by ACR [25] were recruited into this investigator initiated multicenter study between March 2003 and April 2005. NEO-RACo study is a prospective 5 year trial, with extension to 10 years. Follow-up was completed in 2015 after which secondary endpoint analyses were performed. Patients were treated with an intensified FIN-RACo combination including sulphasalazine (up to a maximum of 2 g/day), methotrexate (up to a maximum of 25 mg/week), hydroxychloroquine (35 mg/kg/week), and prednisolone (7.5 mg/day) and double blindly randomized to receive in allocation ratio 1:1 infusions of either placebo (solvent without active drug) or infliximab (3 mg/kg), at weeks 4, 6, 10, 18 and 26. The patients were stratified according to seropositivity (rheumatoid factor defined in a local accredited laboratory). The randomisation was performed centrally by an external laboratory not participating in the clinical trial in blocks of 10 patients. As part of the protocol, all inflamed joints were actively treated with intra-articular injections of glucocorticoids. After 24 months, if the patient was in remission, prednisolone and DMARDs were gradually tapered off with a predefined protocol or in the case of non-remission medication was modified according to the judgment of the treating rheumatologist aiming at strict remission.

The DAS28 score (ESR) was used to evaluate disease activity [19]. The modified Sharp-van der Heijde score was used to evaluate radiologic score [26]. The patient selection criteria as well as the treatment protocol, outcomes and follow-up have been described earlier [20–23].

The study was conducted according to the Declaration of Helsinki. All patients gave informed written consent. The study protocol was approved by the ethics committee of the Hospital District of Helsinki and Uusimaa (statement number 676/E5/02, date January 14, 2002) and registered at Helsinki University Central Hospital HUCH Study Register (date April 29, 2003). The study was registered at the Clinical Trials Database (https://clinicaltrials.gov/ct2/show/NCT00908089?term=NCT00908089&rank=1) in 2009, soon after the revision of the Declaration of Helsinki in 2008, which promoted trial registration in a publicly accessible database. The authors confirm that all ongoing and related trials for this drug/intervention are registered.

### Plasma samples and ELISA

**Fig 1:**
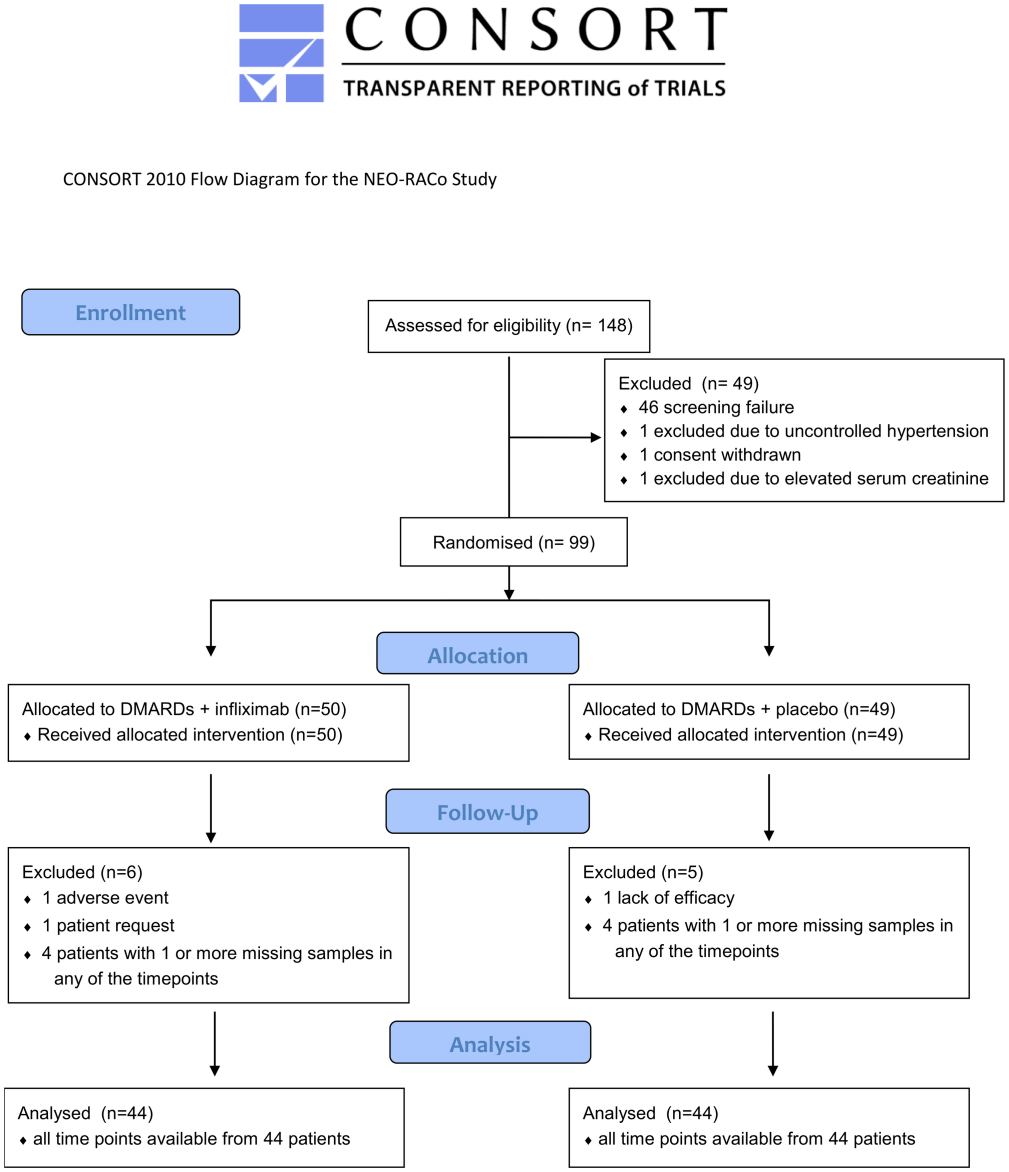
CONSORT flow diagram for the NEO-RACo study.

Plasma samples were collected at weeks 0, 4, 10, 18, 26 (all time points were available from 88 patients, 60 females, Fig 1) and kept at -80°C until assayed. The concentrations of YKL-40 and matrix metalloproteinase-3 (MMP-3) (R&D Systems, Minneapolis, MN, USA), and interleukin-6 (IL-6) (eBioScience Inc., San Diego, CA, USA) were measured by ELISA following the instructions of the manufacturer.

### Statistics

Results are expressed as medians with interquartile ranges (IQRs) or means with standard deviations (SDs) or 95% confidence intervals (95% CIs). Statistical comparisons between the groups were carried out by using t-test, bootstrap type t test (5000 replications) or chi-square test, when appropriate. Analysis of repeated data was carried out by using generalizing estimating equations (GEE) with the unstructured correlation structure. Generalized estimating equations were developed as an extension of the general linear model (e.g. OLS regression analysis) to analyze longitudinal and other correlated data. In longitudinal GEE analyses, DAS28 and its components were used as outcome variables, YKL-40 and time as covariates and the model was adjusted with age, gender, rheumatoid factor positivity and treatment. In the case of violation of the assumptions (e.g. non-normality), a bootstrap-type test was used (5 000 replications). Area under the curve (AUC) was calculated with the trapezoidal method over the time points from 0 to 26 weeks. A possible non-linear relationships between YKL40(AUC) and DAS28(AUC) and IL-6(AUC) was assessed by using regression models with quadratic term. Correlation coefficients were calculated by the Spearman method. The normality of the variables was tested by using the Shapiro-Wilk W test. STATA software (version 13.1, StataCorp, LP, Texas, USA) was used in the statistical analysis.

## Results

**Fig 2:**
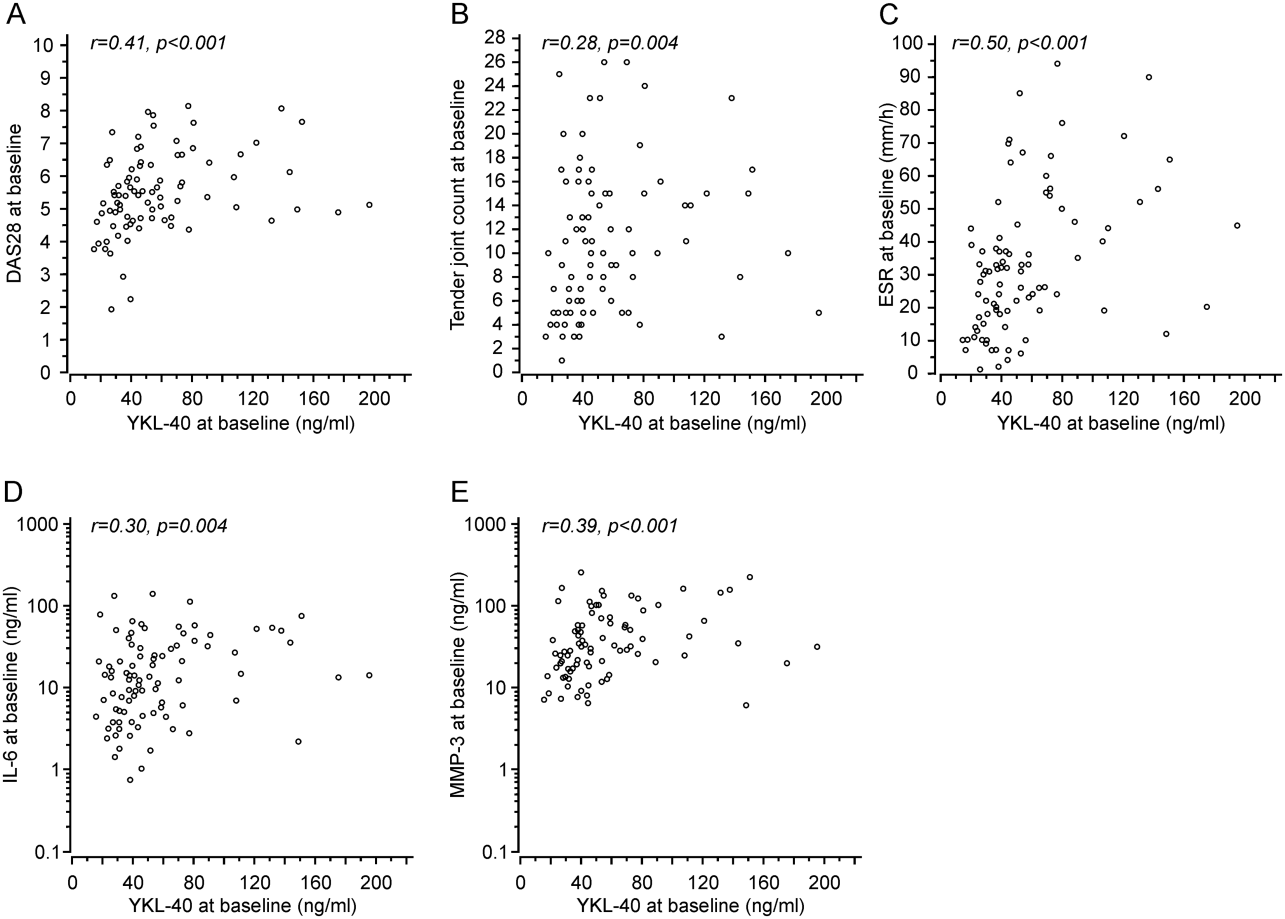
YKL-40 was associated with disease activity, IL-6 and MMP-3 in the DMARD-naïve RA patients. The figure shows scatter plots of YKL-40 levels with disease activity measured as DAS28 (A), tender joint count (B), erythrocyte sedimentation rate (C), IL-6 (D), and MMP-3 (E) at the baseline. Correlation coefficients were calculated by the Spearman method. YKL-40 showed positive correlation to DAS28, tender joint count, ESR, IL-6 and MMP-3 at the baseline.

At the baseline, YKL-40 concentration in the DMARD-naïve RA patients was 57 ± 37 ng/ml (mean ± SD, n = 88) and there was no differences between females and males (55 ± 37 ng/ml, n = 60 vs 61 ± 38 ng/ml, p = 0.43). Characteristics of the patients are shown in Table 1. The levels of YKL-40 under baseline conditions correlated positively with the disease activity measured as DAS28 score (r = 0.41, p<0.001, Fig 2A). When the DAS28 components were assessed separately, the baseline YKL-40 correlated positively with the count of tender joints (r = 0.28, p = 0.004, Fig 2B) and ESR (r = 0.50, p<0.001, Fig 2C), but there was no correlation with the count of swollen joints or patient´s global assessment of general health. Proinflammatory cytokine IL-6 as well as MMP-3 showed a positive correlation with YKL-40 at the baseline (r = 0.30, p = 0.004, Fig 2D and r = 0.39, p<0.001, Fig 2E, respectively).

**Table 1:** The baseline characteristics of the patients.

Characteristic	The initial randomization group		P-value	All
	FIN-RACo + Infliximab (n = 44)	FIN-RACo + Placebo (n = 44)		(n = 88)
Demographic data at baseline				
Female, no (%)	32 (73)	28 (64)	0.41	60 (68)
Age (years), mean ± SD years	47 ± 9	44 ± 11	0.22	46 ± 10)
Duration of symptoms (months), median (IQR)	4 (2, 5)	4 (3, 6)	0.54	4 (2, 6)
Rheumatoid factor present (%)	35 (80)	32 (73)	0.56	67 (76)
Measures of disease activity at baseline				
Swollen joint count, mean ± SD	14 ± 5	16 ± 8	0.33	15 ± 6
Tender joint count, mean ± SD	18 ± 9	21 ± 11	0.10	20 ± 10
Erythrocyte sedimentation rate (mm/h), mean ± SD	33 ± 20	33 ± 23	0.84	33 ± 21
Patient’s global assessment (VAS, mm), mean ± SD	49 ± 25	47 ± 28	0.71	48 ± 26
Pain (VAS, mm), mean ± SD	52 ± 28	51 ± 28	0.79	52 ± 27
Physician’s global assessment (VAS, mm), mean ± SD	46 ± 20	53 ± 20	0.12	50 ± 20
DAS28, mean ± SD	5.4 ± 1.0	5.6 ±1.4	0.66	5.5 ± 1.2
Physical function (HAQ), mean ± SD	1.0 ± 0.6	0.9 ± 0.7	0.47	0.9 ± 0.7
Radiography at baseline				
Erosion score*, mean ± SD	2.5 ± 7.1	1.9 ± 4.4	0.66	2.2 ± 5.9
Narrowing score*, mean ± SD	0.5 ± 1.6	0.3 ± 0.7	0.48	0.4 ± 1.2
Total score, mean ± SD	3.0 ± 8.3	2.1 ± 4.7	0.62	2.6 ± 6.7

Statistical comparison between the groups was performed by t-test, bootstrap-type t-test (5000 replications), or chi-square test, when appropriate.

* Radiologic score by modified Sharp-van der Heijde score.

The patients received a combination of sulphasalazine, methotrexate, hydroxychloroquine and low dose prednisolone for the first four weeks following the intensified FIN-RACo combination treatment strategy [24]. Subsequently, the patients were randomized to receive infliximab or placebo, which was added on the csDMARD combination treatment for the further 22 weeks.

**Fig 3:**
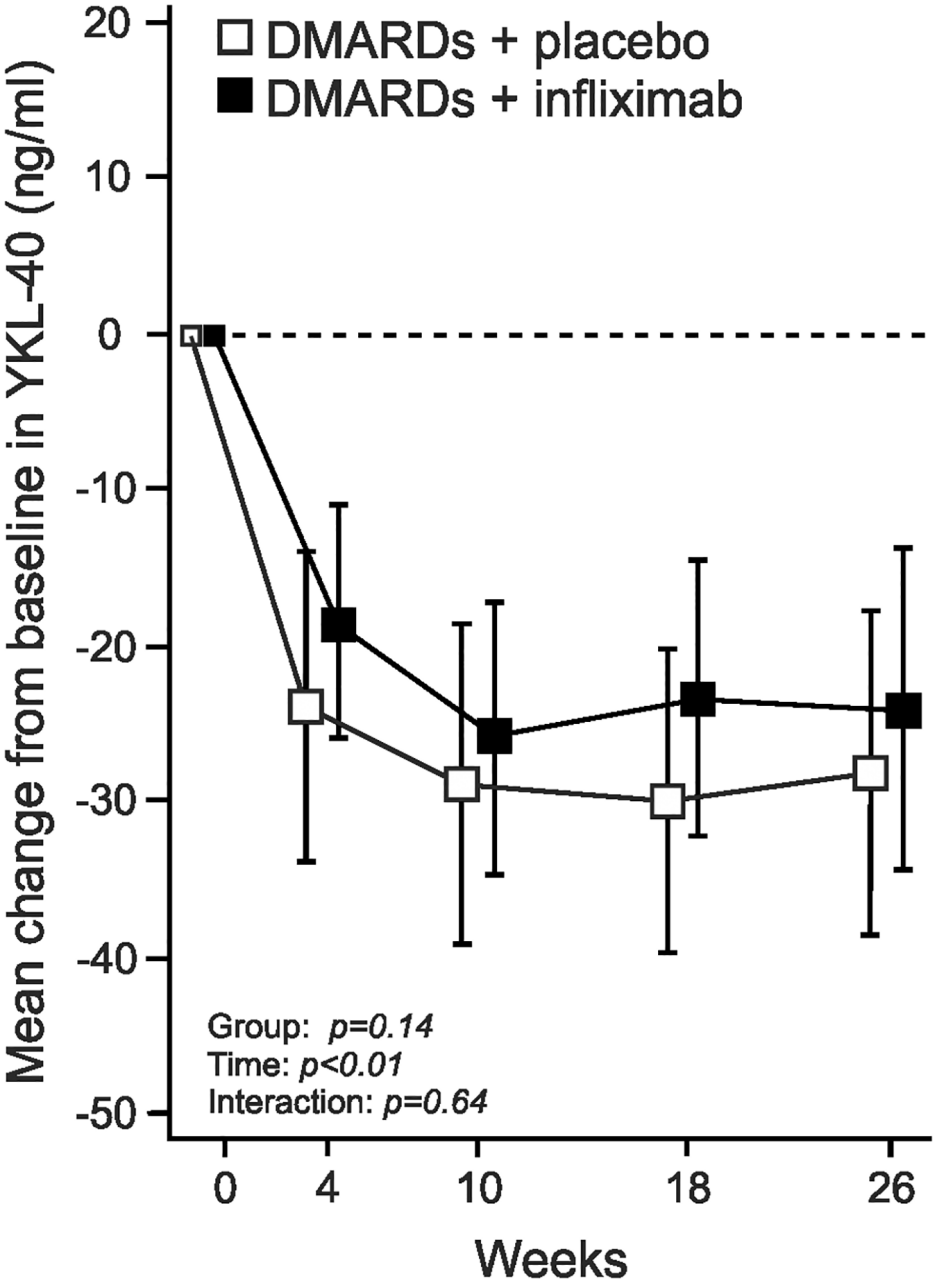
YKL-40 levels decreased during the intensive anti-rheumatic treatment with a combination of csDMARDs. The figure shows the mean change in plasma YKL-40 levels in 88 patients in the NEO-RACo -study treated with a combination of sulphasalazine, methotrexate, hydroxychloroquine and low dose prednisolone for the first four weeks, and thereafter randomized to receive either placebo infusions (Fin-RACo + Pla) or infliximab infusions (Fin-RACo + INFL) added on the csDMARD combination for another 22 weeks. YKL-40 levels decreased significantly (p<0.001) during the first four weeks of treatment with csDMARDs; when infliximab (or placebo) was added to the treatment there was a minor further decrease (groups combined, p = 0.031) but no difference between placebo and infliximab treatment groups was found. The change is presented as ng/ml, mean ± 95% CI, n = 88.

During the first four weeks of treatment with the csDMARD combination, YKL-40 levels decreased significantly (p<0.001, Fig 3). This decrease in YKL-40 levels showed a positive correlation to the change in IL-6 (r = 0.38, 95% CI: 0.18, 0.54) as well as the change in MMP-3 levels (r = 0.42, 95% CI: 0.23, 0.58) from baseline to four weeks; whereas no correlation to the change in DAS28 was found.

During the following 22 weeks of treatment with csDMARD ± infliximab, YKL-40 levels showed a minor further decrease (groups combined: -5.0 ng/ml, 95%CI: -9.6 to -0.5, p = 0.031). However, there was no difference in YKL-40 levels between placebo and infliximab treated groups over time (Fig 3). The result together suggest that decrease in YKL-40 levels is related to the antirheumatic effect of DMARDs, not to infliximab treatment per se.

**Fig 4:**
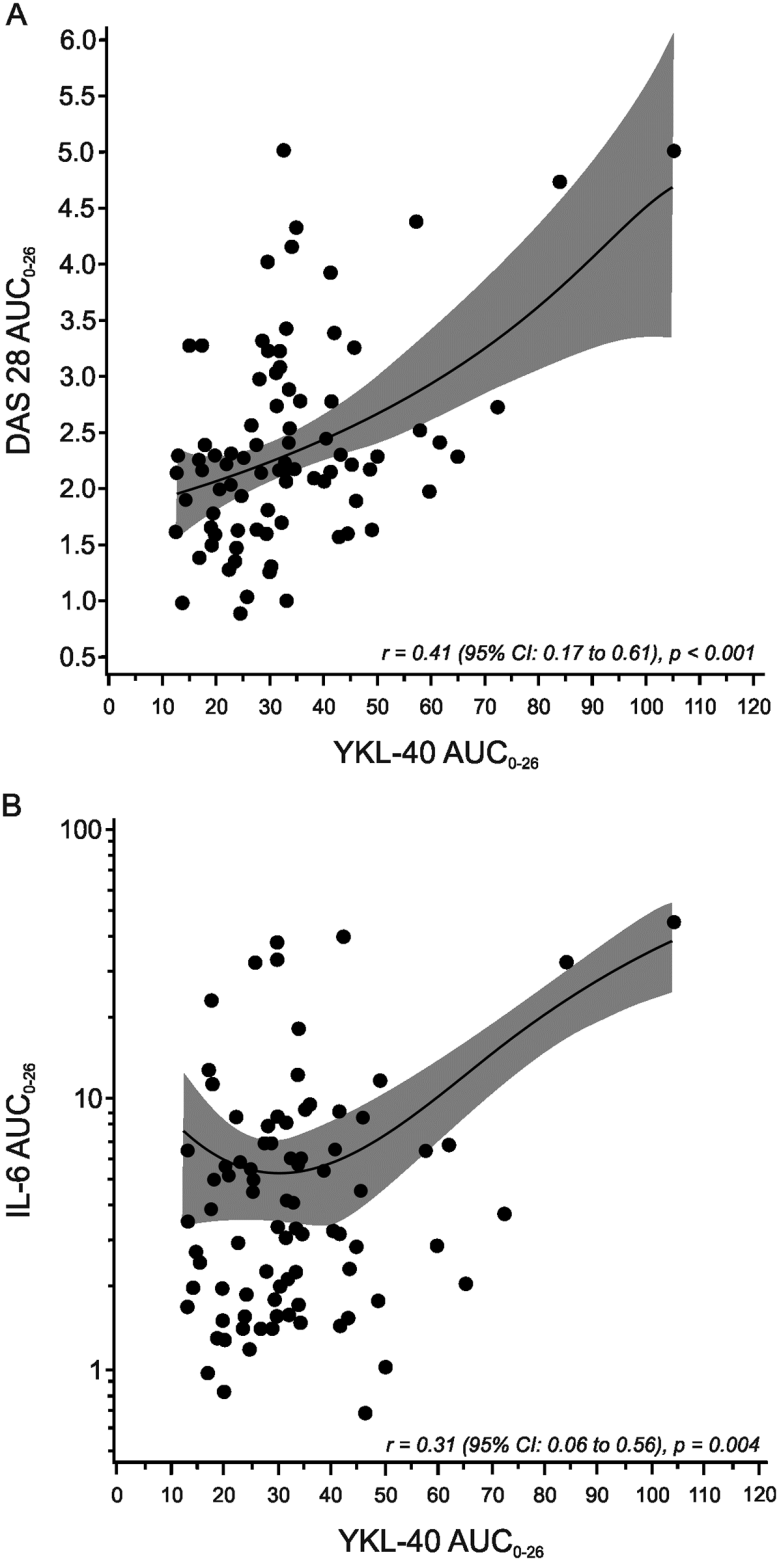
YKL-40 was associated with disease activity and inflammation during intensive treatment with DMARDs. The figure shows quadratic relationships of YKL-40 with the disease activity measured as DAS28 (A) and with the inflammatory cytokine IL-6 (B) during the 26 weeks of treatment. The patients were treated for the first four weeks with a combination of sulphasalazine, methotrexate, hydroxychloroquine and low dose prednisolone, and thereafter randomized to receive either placebo infusions or infliximab infusions added to the treatment for another 22 weeks. Measurements were carried out at weeks 0, 4, 10, 18, and 26 during the treatment and area under the curve analysis over time (AUC_0-26 weeks_) adjusted for age, gender and RF positivity was used. Gray-shaded areas represent 95% confidence intervals around the mean. YKL-40 AUC_0-26 weeks_ showed a statistically significant correlation to DAS28 AUC_0-26 weeks_ and IL-6 AUC_0-26 weeks_.

To assess the associations of YKL-40 with disease activity over time (during the 26 weeks of treatment), area under curve (AUC) analysis adjusted for age, gender and rheumatoid factor (RF) positivity was used. A significant correlation between DAS28 AUC_0-26 weeks_ and YKL-40 AUC_0-26 weeks_ (r = 0.41, p<0.001) was found as shown in Fig 4A. Similarly, during the 26 weeks’ treatment, YKL-40 AUC_0-26 weeks_ showed a significant positive correlation to ESR AUC_0-26 weeks_ (r = 0.56, p<0.001), PGA AUC_0-26 weeks_ (r = 0.23, p = 0.035) as well as to IL-6 AUC_0-26 weeks_ (r = 0.31, p = 0.004, Fig 4B). These results were confirmed in longitudinal generalized estimating equation model (GEE, adjusted for treatment group as well as for age, gender and rheumatoid factor) where YKL-40 showed a significant independent association with DAS28, and associated most potently with ESR and PGA when the individual measures of this variable were analyzed (Table 2).

**Table 2:** Generalized estimating equations models for the effect of YKL40, time and interaction in measures of disease activity from baseline to 26 weeks.

	DAS28		TJC		SJC		ESR		PGA	
	B (95% CI)	p-value	B (95% CI)	p-value	B (95% CI)	p-value	B (95% CI)	p-value	B (95% CI)	p-value
YKL-40	0.03 (0.02 to 0.04)	<0.001	0.08 (0.04 to 0.12)	<0.001	0.07 (0.03 to 0.10)	<0.001	0.32 (0.20 to 0.44)	<0.001	0.29 (0.15 to 0.43)	<0.001
Time	-0.10 (-0.12 to -0.08)	<0.001	-0.17 (-0.25 to -0.09)	<0.001	-0.16 (-0.22 to -0.10)	<0.001	-0.31 (-0.52 to -0.10)	0.004	-0.84 (-1.17 to -0.51)	<0.001
TimexYKL-40		0.24		<0.001		0.004		0.017		0.073

Models were adjusted using age, gender, rheumatoid factor positivity and treatment. DAS28—disease activity score in 28 joints, TJC—tender joint count, SJC—swollen joint count, ESR—erythrocyte sedimentation rate, PGA—patient´s global assessment.

## Discussion

In the present study, we found that high YKL-40 levels were associated with more severe inflammation and with disease activity in DMARD naïve patients with early RA, as well as in the same patients during 26 weeks’ intensive anti-rheumatic treatment. YKL-40 is thus proposed as a potential biomarker of disease activity and inflammation not only in early treatment naïve RA, but also during active drug treatment aiming for remission.

YKL-40 is present in RA joints and its concentrations in synovial fluid are higher than those in serum indicating intra-articular synthesis of the glycoprotein [7,11]. Possible intra-articular cell types producing YKL-40 are fibroblast-like synovial cells, macrophages, neutrophils and chondrocytes [1,11,27]. Hypomethylated DNA loci of the YKL-40 gene were recently detected in synovial cells from RA patients and this epigenetic change was presented to lead to the overexpression of the gene [28]. Also, peripheral blood derived CD16 positive mononuclear cells with a macrophage phenotype infiltrating into the synovium are another potential cellular source of YKL-40 in RA [15]. In addition, lymphocytes may produce YKL-40: Recent study by Tanaka et al. using glucose-6-phospatase isomerase immunized mice as a model of RA, showed overexpression of YKL-40 in splenic regulatory T cells [17].

The cellular effects of YKL-40 are not, however, known very well. It may be that YKL-40 itself is not the functional effector molecule but a precursor. To support that assumption, YKL-40 derived peptides have been shown to induce T-cell proliferation in RA patients [2]. Antigen presenting cells (APCs) from RA synovial fluid were reported to process YKL-40 and present the decay peptides to T-cells [6]. This was associated with the RA-related HLA-DR4 phenotype, and especially the peptide 259–271 was found to induce T-cell proliferation [2,6,29]. Also, the immune response was shifted towards the proinflammatory Th1 type reaction by YKL-40 in patients with RA, but not in healthy controls [5]. Further, T-lymphocytes with RA-associated HLA-DR4 alleles were reported to produce high amounts of interferon-γ and TNF-α when re-exposed to YKL-40 derived peptides, while the production of these cytokines in T-cells with HLA-alleles non-associated to RA was very low [29]. Moreover, complexes of MHC and YKL-40 derived peptides were detected in synovium samples from RA patients and found to associate with characteristic features of rheumatoid synovitis [30]. Also, a positive association between YKL-40 expression in synovial mononuclear cells and joint destruction was reported [15].

There is substantial variability in circulating YKL-40 levels between RA patients, as also found in the present study. In addition to inflammatory joint diseases, increased levels of YKL-40 have been found in other diseases characterized by inflammation and tissue remodeling like asthma, COPD, liver fibrosis, and cancer [1,27]. In addition, certain single nucleotide polymorphisms (SNPs) in the YKL-40 gene have been studied: Nielsen et al. investigated the SNPs in the YKL-40 gene in RA patients and found SNPs that were associated with increased levels of serum YKL-40 but not with RF positivity [31]. However, the frequencies of the investigated SNPs did not differ between RA patients and controls in that study or in the study by Srivastava et al. [31,32].

In the present study, YKL-40 correlated with inflammatory markers IL-6 and ESR both at baseline and during active treatment. To support our data, previous studies based on analysis in a single time point, serum YKL-40 levels have also been shown to associate with ESR and CRP as well as the proinflammatory cytokine IL-6 in RA patients [2,10,33]. The present study extends the previous data by showing that when assessed over time and based on AUC measurements during 26 weeks’ follow-up, YKL-40 correlated with disease activity and inflammation also during the intensive treatment with a combination of DMARDs and infliximab.

YKL-40 correlated also with the disease activity as measured by DAS28 score, at the baseline and during active anti-rheumatic treatment. Similarly to our results, serum YKL-40 was reported to be associated with DAS28 at baseline in DMARD-naïve patients in the study by Knudsen et al. [12] and in a single measurement during DMARD monotherapy reported by Bakker et al. [34]. Also, Kazakova et al., who studied RA patients with active disease despite of on-going DMARD treatment, found a positive correlation between serum YKL-40 and synovial thickening and vascularization depicting synovial inflammation, although no correlation between serum YKL-40 and DAS28 was reported [13]. There are also two other studies based on single measurements of serum YKL-40 during DMARD monotherapy which failed to find correlation between circulating YKL-40 concentrations and the number of swollen or painful joints [8,33]. While the studies by Johansen et al. [9,35] and Peltomaa et al. [10] in addition to the previously mentioned study by Bakker et al [34], which followed early RA patients receiving DMARD monotherapy, showed a positive correlation of the swollen joint count to YKL-40 supporting the present findings.

In the present study, baseline YKL-40 correlated positively also with MMP-3. MMP-3 has been reported to be associated with the disease activity and to predict radiological progression in RA [36]. Previously, we have shown that YKL-40 correlates positively with MMP-3 in synovial fluid and cartilage of OA patients [27].

In the present study, YKL-40 levels decreased significantly during active anti-rheumatic treatment based on intensified Finnish Rheumatoid Arthritis combination (FIN-RACo) therapy [24]. The FIN-RACo regimen consists of a combination of sulphasalazine, methotrexate, hydroxychloroquine and low dose prednisolone supplemented with active treatment of inflamed joints with intra-articular glucocorticoids. The detected decrease in YKL-40 levels reflects the anti-inflammatory effect achieved with the active treatment, which results in excellent sustained clinical results and minimal radiological progression of the disease in long-term follow-up as recently reported by us [20,21]. Johansen et al. [35] reported that YKL-40 levels decreased more rapidly in patients receiving high dose prednisolone together with a DMARD than is patients treated with a DMARD only; in that study, it was also found that methotrexate decreased YKL-40 levels more rapidly than other DMARDs. In addition, intra-articular glucocorticoid injections have been shown to decrease serum YKL-40 levels rapidly and the levels increased when the joint inflammation regained [11].

When the TNF-α antagonist infliximab was added to the intensive csDMARD combination therapy in the present patients, it did not cause any further decrease in the YKL-40 levels. This is presumably because the patients were already on the active FIN-RACo-combination treatment and had an excellent treatment response. The absent effect of infliximab on YKL-40 levels is also in agreement with the previous study, in which intravenously given TNF-α did not have any effect on plasma YKL-40 levels in healthy volunteers [37]. There are also studies in which TNF-α antagonists have been shown to decrease YKL-40 levels in RA patients [38–40]. In those studies, however, csDMARDS and glucocorticoids were not used as actively as in the present study where FIN-RACo combination treatment was supplemented with infliximab in a randomized and placebo-controlled manner. The decrease in the YKL-40 levels is thus likely due to anti-inflammatory treatment response itself rather than a direct TNF-α antagonist effect on the YKL-40 levels. This is supported by the finding reported in patients with spondyloarthritis (SpA) treated with TNF-α antagonist: serum YKL-40 concentrations decreased during successful anti-TNF-α treatment whereas YKL-40 levels remained high in non-responding patients [41].

In the present study we investigated the potential of YKL-40 as a single biomarker in early RA. This is advantageous over multibiomarker disease activity tests (MBDA) not only because of its inexpensiveness but also as biologicals/tsDMARDs have been shown to have compensatory increasing effects on the levels of the individual cytokines/mediators included in the MBDA set which may result in underestimation of clinical responses to the treatments. For instance, in the ACT-RAY study, blocking of IL-6 receptor with tocilizumab increased IL-6 levels over 400% [42]. In another study, RA patients treated with a janus kinase 1/3 signaling inhibitor tofacitinib had significantly increased leptin levels [43]. The increasing effect of tofacitinib on leptin levels may be a compensatory mechanism as leptin signals through leptin receptor (ObR) activating JAK/STAT pathway. Most recently, the Vectra DA MBDA-test failed to reflect clinical disease activity in the AMPLE trial during the treatment with abatacept, an inhibitor of T-cell activation, or adalimumab, an anti-TNF agent [44].

The present results, together with the previously published data, suggest that glycoprotein YKL-40 may be used as a biomarker of disease activity in RA, both in the early disease in treatment naïve patients and during active treatment with DMARD combination with or without infliximab.

## Supporting information

S1 File CONSORT 2010 checklist for the NEO-RACo study. (DOC)

S2 File Original protocol for the NEO-RACo study (2001-12-27 tnf-combi-rev.rtf). (RTF)
