# Supplementary material for: Glycoprotein YKL-40: A potential biomarker of disease activity in rheumatoid arthritis during intensive treatment with csDMARDs and infliximab. Evidence from the randomised controlled NEO-RACo trial
Source: PLoS One. 2017 Aug 25;12(8):e0183294. doi: 10.1371/journal.pone.0183294 (PMC5571914; doi:10.1371/journal.pone.0183294)
Supplement: S2 File — (RTF) [file pone.0183294.s002.rtf]

					Version 1/27.12.2001		
STUDY PROTOCOL

USE OF TNF-BLOCKING THERAPY IN COMBINATION WITH DMARDS IN PATIENTS WITH EARLY RHEUMATOID ARTHRITIS

A 5- year prospective randomised study 

1. Introduction

Rheumatoid arthritis (RA) is a chronic disease with varying grades of severity. In most cases, the chronic inflammation runs its own course and leads to permanent joint destruction. The development of joint destruction detected as erosions by radiology starts early during the first 2 years from the onset of symptoms  (Larsen and Thoen 1987). We have previously observed that a third of RA patients have radiological changes during the first year (Paimela et al. 1991). This has lead into discussions about the significance of early treatment of patients; by early treatment, best when initiated before permanent lesions have started to develop, we probably would have the best chances to preserve joint function. There is controlled evidence that at least short courses of treatment with antirheumatic drugs (DMARDs) can diminish inflammation measured by laboratory (ESR and CRP) and by clinical variables (number of swollen and tender joints) and can decrease functional disability (Paulus et al. 1990). Despite of the good clinical response, there is often a progressive joint destruction, as observed by us (Paimela et al. 1991, Möttönen et al. 1996) and by others. There is, however, increasing evidence that early and effective suppression of inflammation can retard the radiological progression in patients with early RA (Stenger et al. 1998).

Due to individual responses, the therapeutic outcome of patients with RA treated with a single disease modifying drug (DMARD) is hard to predict. In addition, the therapeutic response evaluated by the ACR criteria (Felson et al. 1995) is only modest: ACR20% response is observed in 23-65%, ACR50% response in 9-35%, and ACR70% in 1-9% of the patients, with the highest responses usually observed in patients on methotrexate (Felson et al. 1998). Remissions, according to the ACR preliminary criteria (Pinals et al. 1981) are observed in about 20% of the patients, but usually only transiently. By individual tailoring of the treatment with frequent changes and use of combinations the response rates can be increased with remissions in about 32% of the patients (Möttönen et al. 1996). 

Combinations of DMARDs have been applied recently in the treatment of RA. Many of the combinations have only week positive efficacy compared with monotherapy. However, the triple therapy with methotrexate, sulphasalazine, and hydroxy-chloroquine with either prednisolone, if clinically indicated, in chronic RA (O'Dell et al. 1996), or as an essential part of the combination in early RA (Möttönen et al. 1999) has proved to be highly effective with >50% response observed in about 75% of the patients (O'Dell et al. 1996; Möttönen et al. 1999). In addition, patients treated with the triple therapy with prednisolone, had more often remissions at 2-year time point (37%) compared with patients on monotherapy (18%; Möttönen et al. 1999). A positive effect on the retardation of radiological progression was also observed.
 
Recently, biological drugs, especially drugs inhibiting the function of TNF (anti-TNF-antibody [infliximab] or soluble TNF receptor fusion protein [etanercept]) have shown to be effective in patients with severe acute or chronic RA. The drugs have a rapid mode of action, with a response observed during the first 2 weeks after subcutaneously administered etanercerpt or after single infusion of infliximab. A combination of infliximab with MTX seem to have additive effect and to prolong the effect of anti-TNF therapy (Maini et al. 1998). The maximum effect is reached by 4-6 months. The responses with anti-TNF therapies have been better than with conventional monotherapy (Maini et al. 1999; Lipsky et al. 2000; Bathon et al. 2000), but are lower than in the latest combination therapy. Recent data (Lipsky et al. 2000, Bathon et al. 2000) have also demonstrated that anti-TNF therapies can even stop the progression of joint destruction.

During the use of TNF blocking agents, the most common adverse events are the local irritation at the injection site (in studies on etanercept) or those associated with the infusion of antibody (headache, nausea, vasovagal reactions, and urticaria). Most of the adverse events can be prevented by extending the infusion time at least to 2 hours. With infliximab, potentially important adverse events can be grouped into a) risk for infection; b) autoimmune manifestations, and c) immunogenicity. 

 In a study on combination of MTX with infliximab (Maini et al. 1998), minor adverse events were more common in patients receiving the combination (82.8%) than in those receiving MTX + placebo (57.1%, p=0.186). Pharyngitis occurred in 6.9%, rhinitis in 6.9%, cough in 5.7%, upper respiratory tract infection in 4.6%, and urinary tract infection in 4.6%. The frequency of serious adverse events were observed in 16% in patients on MTX + infliximab vs. 7 % in those on MTX + placebo. The respecting figures for serious infections were 7% vs. 2% (Maini et al. 1999).  Among 147 000 patients having received infliximab, 70 cases of tuberculosis have recently been reported (Keane et al 2001), which has resulted in an increased awareness and caution to treat patients with a previous history of or contact to subjects with tuberculosis.

Preliminary results with infliximab as monotherapy reported the development of anti-DS-DNA antibodies in about 10% of the patients (see Kavanaugh 1998). About 1% of patients treated with infliximab have developed drug-induced SLE which has disappeared after discontinuation of the therapy (Scheible 2001).

The development of antibodies to the human chimeric anti-TNF antibody (HACA) seems to be depending on the dose of the antibody (higher doses being less immunogenic) and on the use of methotrexate in combination with the antibody. In a study of infliximab 3 mg/kg as a single therapy, HACA developed in 21% of the patients, while such event was observed in only 7% of patients with a combination treatment with infliximab and methotrexate (Maini et al. 1998). 

2. Aim of the study

We want to study, whether early treatment with infliximab for 6 months started parallel with the combination therapy of methotrexate, sulphasalazine, hydroxychloroquine and prednisolone (COMBI) 
1)	can induce quick remission in patients with early RA,
2)	can sustain the remission after 6 months on patients continuing the COMBI treatment, 
3)	can diminish the risk of progression of erosive changes in patients with early RA, and
4)	can diminish the direct and indirect costs of early active RA and retard/diminish the work disability due to the disease.

3. Statistical analysis

3.1 Sample size
The target sample size about 100 patients was calculated on the basis of a previous study (Möttönen et al. 1999), where at the 6 month visit, 25% of patients in combination therapy had remission. With a power of 90% to detect a difference of 30% or greater remission rate in patients receiving  INFLIXIMAB vs. placebo (50% vs. 25%), the size in each group is 50 patients (two-sided =0.05). Similarly, the numbers of patients needed in each treatment arm to detect a 30% difference (=0.05) in the numbers of non-eroded joints at 24 months with a power of 90% was 50 in each treatment arm.

4. Patients

100 patients with early RA will be included in the study. The patients are randomised into COMBI + placebo or into COMBI + INFLIXIMAB. 

4.1 Inclusion criteria
a.	Diagnosis of RA fulfilling the ACR classification criteria for RA (Arnett et al. 1988);
b.	To be able to observe the effect of therapy on work disability, patients within age group of 18-65 years;
c.	Patients not permanently work disabled or retired;
d.	Duration of symptoms < 12 months, and who have not received DMARD previously;
e.	Patients with active disease (see below) 
f.	Previous treatment with prednisolon or equivalent maximum 10 mg/day for a maximum of 1 month;
g.	no oral glucocorticosteroid treatment during the preceding 1 month at entry

4.2 Criteria for active disease at entry
a.	>6 swollen joints (66 joint count)
b.	>6 tender joints (68 joint count)
c.	duration of early morning stiffness >45 min and/or ESR >30 mm/h and/or CRP >20 mg/l

The patients will be stratified according to seropositivity. Seropositive disease is defined in a patient with a positive test in quantitative rheumatoid factor (defined as a value above the normal upper limit for  rheumatoid factor in a local accredited laboratory). The randomisation will be performed centrally by an external laboratory not participating in the trial. The randomisation will be performed in blocks of 20 envelops. 

4.3 	Exclusion criteria
1) 	Previous treatment with DMARDs for RA
2) 	Previous treatment with oral glucocorticoids > 1 month or >20 mg/day of prednisolone or equivalent because of RA
3) 	Use of oral prednisolone during previous 3 months before inclusion in the study
4) 	Minimum of 30 days from previous intra-articular injection with glucocorticosteroids
5) 	Allergy to sulphonamides
6) 	Allergy to acetylsalicylic acid
7) 	Allergy to methotrexate
8) 	Allergy to antimalarials
9) 	Previous treatment with biologicals
10) 	Serum creatinine value >upper limit of normal
11) 	Serum transaminase levels >2x upper limit of normal
12) 	Known/previous malignancy excluding basalioma or in situ cervical cancer >5 years previously
13) 	Cardiac failure 
14) 	Previous history of tuberculosis and/or exposition to tuberculosis and/or typical changes of previous/active tuberculosis in chest radiology
15) 	Active infection
16) 	Pregnancy
17) 	Leukopenia (WBC <4 x 10^9^/l)
18) 	Thrombocytopenia (platelets <100 x 109/l)
19) 	Active peptic ulcer
20) 	Type I or type II diabetes under  poor control
21) 	Heavy use of alcohol
22) 	Fertile women not practising contraception or who are planning pregnancy
23) 	Male patients wishing to have children during the therapy
24) 	Other autoimmune rheumatic disease
25) 	Other chronic disease which judged by the physician could influence the patient's compliance or intervene the study course
26) 	Patient is not cooperative

5 Drugs

All patients will start with a combination of methotrexate (MTX) + sulfasalazine (SASP) + hydroxychloroquine (HCQ) + prednisone/prednisolone (PRED) orally (see 5.2).

In addition, the patients will be randomised to a treatment with infliximab (anti-TNF-monoclonal antibody) 3 mg/kg, or with similar placebo (solvent without active drug). The treatment will be started after 1 month on the combination treatment with intravenous infusions at 4, 6,10, 18 and 26 weeks.


5.1 Patient evaluation

The patients will be evaluated clinically at week 0, 4, 6, 10, 14, 18, 22 and 26 (at the day of infusion, prior to the infusion) and at months 8, 10, 12, 15, 18, 21, and 24 and at years 3, 4 and 5. The evaluation includes clinical examination, patient evaluation of pain (VAS), patient's global assessment (VAS), physician's global assessment (VAS) and HAQ. Immediately prior to the infusion and at months 8, 10, 12, 15, 18, 21, and 24 and at years 3, 4 and 5, laboratory tests for safety and efficacy will be drawn. 

5.2 Treatment strategy

Both treatment arms include combination therapy with MTX, SASP, HCQ and PRED. In addition, the patients will be randomised to receive INFLIXIMAB/ PLACEBO infusions during the first 6 months (weeks 4, 6, 10, 18, and 26). By 26 weeks, the infusions will be stopped, and the COMBI will be continued in both arms. 

Dose adjustment of individual DMARDs

Initial dose of MTX 10 mg/week, increased by next 4 weeks to 15 mg/week. At week 10, if the patient still has active disease defined by the presence of at least 1 swollen joint, the dose will be increased to 20 mg/week. At week 14, if the patient still has at least 1 swollen joint, the dose is increased to 25 mg/week. The dosage of 25 mg/week if the maximum tolerated dose is thereafter continued. Due to adverse events, the MTX can be switched to intramuscular (im) or subcutaneous (sc), with similar dose. 

The clinical evaluation with dose adjustment will be performed at week 4 (prior to infusion), week 10 (prior to infusion), week 14, and week 18 (prior to infusion). Folic acid is added in the treatment of all the patients, with 5 mg/week.

Initial dose of SASP is 1 g/day for 2 weeks, thereafter the dose is increased to 2 g/day, or the maximum dose the patient tolerates (1-2 g/day). 

HCQ will be administered evenly though a week, with a weekly dose of 35 mg/kg/week. After a treatment for 1 year, a pause for 1 month during the summertime will be used.

The dose of PRED is fixed to 7.5 mg/day during the first 2 years. Following recent guidelines for arthritis patients treated with long-term glucocorticoid therapy with prednisolone equivalent of > 5 mg/day, the patients should receive calcium and vitamin D supplementation (ACR ad hoc committee 2001) and bone mineral density should be measured before the therapy and if normal, follow-up mesurements should be performed either annually or biannually (ACR ad hoc committee 2001). Therefore, all patients should receive calcium and vitamin D supplementation (1000 mg calcium/daily and 800 IU vitamin D3/daily).

The evaluation of the dose increments is based on the achievement remission. Remission is recorded at each visit. The aim is to reach remission. If the patient has been on maximal tolerated doses of MTX, SASP, HCQ and PRED at least 3 months, the drug(s) will be changed with the purpose to increase the therapeutic response. 
MTX, SASP or HCQ will be substituted to another single DMARD according to the “sawtooth“ method according to the following order:

1.	MTX substituted with azathioprine (starting dose 50 mg/day, max. 2.5 mg/kg/day)
2.	SASP substituted with cyclosporin  (starting dose 2.5 mg/kg/day, max. 4 mg/kg/day) 
3.	HCQ substituted with auranofin (starting dose 6 mg/day, max 9 mg/day)
In the case of allergies/intolerance to the above-mentioned substitution(s), other DMARDs can be used if needed as follows:
1.	Injectable gold (Myocrisin, starting dose 10 mg im., increased to 50 mg/month) (after week 26)
2.	Cyclosporin (starting dose 2.5 mg/kg/day, max. 4 mg/kg/day)

The combination should always include at least one cytostatic or immunomodulating drug (MTX, azathioprine or cyclosporin).

Dose adjustments in the case of sustained remission

If the patient has achieved remission or at least > ACR50% response after dose adjustments/drug substitutions, the combination will be continued up till 24 month visit. If the patient is in remission at month 24, the dose reduction of individual drugs in the COMBI can be started in the following order:
1.	PRED dose decreased 2.5 mg/day/3 months, followed by additional decrease of 2.5 mg/day for 3 months, followed by an additional 2.5 mg/day for 3 months, followed by an additional 2.5 mg/day for 3 months. If the patient is still in remission after 3 months (= at 3 year visit), PRED can be tapered off. 
2.	If the PRED has been tapered off and the patient is still in remission for 3 months, the dose of SASP can be reduced by 500 mg/day for 3 months. If the patient is still in remission, the dose can be further reduced by 500 mg/day for every 3 months, assuming that the patient is in remission.
3.	If SASP has been tapered off and the patient is still in remission, the dose of MTX can be reduced by 2.5 mg/week/3 months, If the patient is still in remission, the dose can be further reduced by 32.5 mg/week/by each 3 months, assuming that the patient is still in remission.
4.	If the patient loses remission, the previous drug and dose should be added in the treatment protocol.

Dose in the case that patient has a sustained response > ACR50%

If the patient has a sustained good response but is not in remission, the maximum tolerated doses in COMBI will be continued. ACR50% improvement criteria (Felson et al. 1995) applied as follows:
·	>50% improvement in tender joint count  (68 joints)
·	>50% improvement in swollen joint count (66 joints)
·	>50% improvement in 3 out of following 5:
·	patient pain assessment (10 cm VAS)
·	patient global assessment (10 cm VAS)
·	physician global assessment (10 cm VAS)
·	patient self-assessed disability (HAQ)
·	acute phase reactant (ESR or CRP)

5.3 Treatment failure

If, after dose and drug adjustments,  the patient is a non-responder (<ACR50% at maximal combination after individual substitutions) at 2 consecutive visits, the patient is regarded as a treatment failure, and the therapy is open, including the possibility to use anti-TNF blocking agents. However, the code for previous infliximab/placebo therapy is not opened. Treatments/combinations can be applied freely, but he/she will continue in the study up till 5 year evaluation, with information collected in the case report files for the whole 5-year period according to the protocol with laboratory and radiological analysis as primarily planned.

6 Monitoring safety

Laboratory safety variables along the routine guidelines include WBC + differential, platelets, haemoglobin, serum creatinine, serum ASAT, ALAT, alkaline phosphatase, urine sediment. Tests will be performed at weeks 0, 2, 4, 6, 10, 14, 18, 22 and 26 (at the day of infusion, prior to the infusion) and at months 8, 10, 12, 15, 18, 21 and 24 months and thereafter at 3, 4 and 5 years. If the dose of any MTX or SASP is increased, or a new DMARD is started, additional safety tests will be performed 2 weeks later.

Serum samples for antinuclear antibodies (ANA) and antibodies against double-stranded DNA (DS-DNA antibodies) will be collected at 0, 4, 10, 18, and 26 weeks, and thereafter at 8, 10, 12, 15, 18 and 24 months and thereafter at 3, 4 and 5 years. The sera will be frozen at –20oC and analysed at later phase.

Chest X-ray is studied at entry, and at 1, 2 and 5 years.

7 Monitoring of disease activity

The disease activity will be measured according to the ACR core set of disease activity (Felson et al. 1993), applied as in the following: 
·	tender joint count (68 joints), 
·	swollen joint count (66 joint count), 
·	patient's assessment of pain (10 cm VAS), 
·	patient's global assessment of disease activity (10 cm VAS), 
·	physician's global assessment of disease activity (10 cm VAS),
·	Patient's assessment of physical function (HAQ)
·	Acute-phase reactant value (ESR and CRP)

8 Monitoring radiological progression

Radiology of hands (PA projection) and feet (PA projection) at 0, 12 months and at 2, 3 and 5 years. Radiographs will be read later on by an expert in joint radiology blinded by the clinical data and treatment protocol. Radiology of cervical spine (AP and lateral projection with flexion/extension) will be performed at entry and at 5 years.

9 Monitoring of bone density

Bone density will be measured at entry, and at 2 and 5 years.

10 Monitoring the quality of life 

The quality of life will be recorded by 15D and SF-36 questionnaire at months 0, 6, 12, 18, 24 and thereafter at 3, 4 and 5 years.

11. Pharmacoeconomic evaluation

Both direct costs (costs for in-patient, out-patient and emergency care) and indirect costs (sick leaves, costs due to need of domestic help) and costs due to health service utilization (number of out-patient visits to doctors and to other health-care professionals, out-patient visits, laboratory and other diagnostic procedures, prescription and non-prescrition medications), and assistive devices  during the study. The cumulative data about sick leaves (days and loss of wages and compensation paid by either the employer or the insurance) and date of possible work disability due to RA will be collected.

12 Other studies

Serum  (10 ml) and plasma (10 ml) will be collected and stored at –20oC by months 0, 6, 12, 18, 24 and thereafter at 3, 4 and 5 years for the later analysis of ICTP and cytokines.

10 ml of EDTA blood will be collected and stored at  –20oC at entry for later analysis of HLA antigens and cytokine polymorphism.

13 Statistical treatment

Primary outcome variables:
1)	Remission at 2 years (Pinals et al. 1981);
2)	Number of patients with remission at each visit during the observation period;
3)	Number of patients with >ACR50% response at each visit during the     observation period;
4)	Number of patients with sustained remission from month 3 till the end of the         study;
5)	Radiological changes at 24 months;
6)	Direct and indirect costs at 2 years.

Secondary outcome variables:
1)	Remission at 1 year;
2)	HAQ at 12 months, and at 2, 3, 4 and 5 years;
3)	Cumulative dose of glucocorticoid injections at 2 years;
4)	Cumulative dose of glucocorticoid injections as prednisolone equivalents by 2 years;
5)	Permanent work disability at 2 years and thereafter at 3, 4 and 5 years;
6)	Number of patients with sustained good response (>ACR50%) from month 3         till the end of the study;
7)	Cumulative number of arthroplasties at 5 years;
8)	Pharmacoeconomic analysis of direct and indirect costs at 5 years.

14 Adverse events

All adverse events (AE) will be recorded in the case files. Special attention is paid to the occurrence of infections and malignancies.

The adverse events will be grouped in 3 categories:
1) mild AE
2) moderate AE
3) severe AE

If there is an increase in liver enzyme levels >2x upper limit of normal, the MTX dose is decreased guided by routine praxis. If the liver enzyme levels increase >3 upper limit of normal, MTX and SASP are paused. If the enzyme levels are decreasing and end up with < 2 upper limit of normal, both drugs can be sequentially reintroduced, but with lower doses. In the case of haematological adverse events, MTX and SASP can be interrupted for 2 weeks, and if recovery, instituted sequentially by lower dose, starting with MTX. In the case of severe adverse events, one or both of the DMARDs should be substituted with another DMARD after the recovery.

All adverse events will be recorded in the case report forms as open text. 
1) 	gastrointestinal
2) 	hepatic
3) 	urogenital
4) 	neurological
5) 	cardiovascular
6) 	respiratory
7) 	skin/mucocutaneous
8) 	haematological
9) 	musculoskeletal
10) 	infections
11) 	malignancies
12) 	other, which…………..
In addition, the adverse events will be evaluated in detail by description of the adverse event.

All adverse events will also be characterised as to the severity of the event as follows:
1) 	Mild: symptom observed/reported by the patient, tolerated, does not disturb normal activity
2) 	Moderate: symptom observed/reported by the patient, does interfere with daily activities, but tolerated
3) 	Severe: symptoms interfering with daily activities, drug(s) have to be discontinued or paused, may need additional remedies

All the adverse events have to be reported whether or not they are serious. Serious adverse event is defined as:
1) 	death
2) 	admission to hospital (for any reason)
3) 	malignancy
4) 	severe adverse, potentially life threatening event leading to immediate cessation of therapy and possibly also treatment with other drugs

The course of the adverse event will be recorded in 2 ways:

1) 	the course of the DMARD/infliximab treatment
n	continues normally
n	the dose(s) were diminished
n	the drug(s) are paused
n	the drug(s) are stopped and the component(s) are substituted
2) 	the outcome of the adverse event
n	the adverse event disappeared/the patient recovered
n	the patient received drug(s) treatment for adverse event
n	the adverse event continued but was tolerated
n	the patient discontinued the study
n	the patient died

15 Use of NSAID

NSAID can be used as clinically indicated. All the drugs have to be recorded in the case files.

16 Local corticosteroid injections

Local corticosteroid injections can be used as needed. The joint(s) injected with the dose(s) of glucocorticoids (as equivalent of mg of prednisolone) has to be recorded in the case files.

17 Other medications

All the medications used by the patient have to be recorded in the case files.

Duration of the study
The duration of the study for one patient is 5 years. An interim analysis will be performed at month 12 and 24. 


References

American College of Rheumatology Ad Hoc Committee on Glucocorticoid-Induced Osteoporosis: Recommendations for the prevention and treatment of glucocorticoid-induced osteoporosis. Arthritis Rheum 44: 1496, 2001

Arnett FC, et al: The American Rheumatism Association 1987 revised criteria for the classification of rheumatoid arthritis. Arthritis Rheum 31: 315, 1988

Bathon JM,et al: A comparison of etanercept and methotrexate in patients with early rheumatoid arthritis. N Engl J Med. 343: 1586, 2000

Felson DT, et al: The American college of rheumatology preliminary core set of disease activity measures for rheumatoid arthritis clinical trials. Arthritis Rheum 36: 729, 1993

Felson DT, et al: American College of Rheumatology preliminary definition of improvement in rheumatoid arthritis. Arthritis Rheum 38: 727, 1995

Felson DT, et al: Should improvement in rheumatoid arthritis clinical trials be defined as fifty percent or seventy percent improvement in core set measures, rather than twenty percent? Arthritis Rheum 41: 1564, 1998

Kavanaugh AF: Anti-tumor necrosis factor- monoclonal antibody therapy for rheumatoid arthritis. Rheum Dis Clin North Am 24: 593, 1998

Larsen A, Thoen J: Hand radiography of 200 patients with rheumatoid arthritis repeated after an interval of one year. Scand J Rheumatol 16: 395, 1987

Lipsky PE, et al: Infliximab and methotrexate in the treatment of rheumatoid arthritis. Anti-Tumor Necrosis Factor Trial in Rheumatoid Arthritis with Concomitant Therapy Study Group. N Engl J Med343:1594, 2000

Keane J, et al: Tuberculosis associated with infliximab, a tumor necrosis factor alpha-neutralizing agent. N Engl J Med 345:1098, 2001

Maini RN, et al: Therapeutic efficacy of multiple intravenous infusions of anti-tumor necrosis factor  monoclonal antibody combined with low-dose weekly methotrexate in rheumatoid arthritis. Arthritis Rheum 41: 1552, 1998

Maini R, et al: Infliximab (chimeric anti-tumour necrosis factor  monoclonal antibody) versus placebo in a rheumatoid arthritis patients receiving concomitant methotrexate: a randomised phase III trial. Lancet 354: 1932, 1999

Moreland LW, et al: Treatment of rheumatoid arthritis with a recombinant human tumor necrosis factor receptor (p75)-Fc fusion protein. N Engl J Med 337: 141, 1997

Möttönen T, et al: Outcome in patients with early rheumatoid arthritis treated according to the "saw tooth" strategy. Arthritis Rheum 39: 996, 1996

Möttönen T, et al: Comparison of combination therapy with single-drug therapy in early rheumatoid arthritis: a randomised trial. Lancet 353: 1568, 1999

O'Dell JR, et al: Treatment of rheumatoid arthritis with methotrexate alone, sulfasalazine and hydroxychloroquine, or a combination of all three medications. N Engl J Med 334: 1287, 1996

Paimela L, et al: Serum hyaluronate level as a predictor of radiologic progression in early rheumatoid arthritis. Arthritis Rheum 34: 815, 1991

Paulus HE, et al: Analysis of improvement in individual rheumatoid arthritis patients treated with disease-modifying antirheumatic drugs, based on the findings in patients treated with placebo. Arthritis Rheum 33: 477, 1990

Pinals RS, et al: Preliminary criteria for clinical remission in rheumatoid arthritis. Arthritis Rheum 24: 1308, 1981

Schaible TF:Treatment of inflammatory diseases: safety of long-term use of infliximab. Presse Med  30: 610, 2001

Stenger AAM, et al: Early effective suppression of inflammation in rheumatoid arthritis reduces radiographic progression. Br J Rheumatol 37: 1157. 1998

Weinblatt ME, et al: Low-dose methotrexate compared with auranofin in adult rheumatoid arthritis: a thirty-six-week, double-blind trial. Arthritis Rheum 33: 330, 1990

Tugwell P, et al: Combination therapy with cyclosporine and methotrexate in severe rheumatoid arthritis. N Engl J Med 333: 137, 1995
